# Tubulin is actively exported from the nucleus through the Exportin1/CRM1 pathway

**DOI:** 10.1038/s41598-019-42056-6

**Published:** 2019-04-05

**Authors:** K. Schwarzerová, E. Bellinvia, J. Martinek, L. Sikorová, V. Dostál, L. Libusová, P. Bokvaj, L. Fischer, A. C. Schmit, P. Nick

**Affiliations:** 1 Department of Experimental Plant Biology, Faculty of Science, Charles University, Viničná 5, Prague, Czech Republic; 2 Department of Cell Biology, Faculty of Science, Charles University, Prague, Viničná 7, Czech Republic; 3 Institut de Biologie Moléculaire des Plantes, Centre National de La Recherche Scientifique, Université de Strasbourg, F67084 Strasbourg-cedex, France; 4 Molecular Cell Biology, Botanical Institute, Karlsruhe Institute of Technology (KIT), Fritz-Haber-Weg 4, 76131 Karlsruhe, Germany

## Abstract

Microtubules of all eukaryotic cells are formed by α- and β-tubulin heterodimers. In addition to the well known cytoplasmic tubulins, a subpopulation of tubulin can occur in the nucleus. So far, the potential function of nuclear tubulin has remained elusive. In this work, we show that α- and β-tubulins of various organisms contain multiple conserved nuclear export sequences, which are potential targets of the Exportin 1/CRM1 pathway. We demonstrate exemplarily that these NES motifs are sufficient to mediate export of GFP as model cargo and that this export can be inhibited by leptomycin B, an inhibitor of the Exportin 1/CRM1 pathway. Likewise, leptomycin B causes accumulation of GFP-tagged tubulin in interphase nuclei, in both plant and animal model cells. Our analysis of nuclear tubulin content supports the hypothesis that an important function of nuclear tubulin export is the exclusion of tubulin from interphase nuclei, after being trapped by nuclear envelope reassembly during telophase.

## Introduction

Tubulin is a heterodimeric protein that consists of α- and β-tubulin subunits. The tubulin heterodimer polymerizes to form microtubules, a dynamic cytoskeletal structure conserved in all eukaryotic cells. Besides their conserved role in cell division (mitosis and meiosis), microtubules play crucial roles during cytokinesis and during interphase. In animal cells, microtubules are involved in determination of cell shape and various kinds of cell movements, including different forms of cell locomotion, or intracellular transport of organelles in addition to their role in the segregation of chromosomes. In plant cells, cortical microtubules participate in cell wall synthesis and cell division. In most eukaryotes, microtubules and their subunits, the α/β-tubulin heterodimers, are only found in the cytoplasm; there are no known roles of microtubules or tubulin in the nucleoplasm so far except for eukaryotes exhibiting closed mitosis (for review see^1^). However, the presence of α- and β-tubulin has been also noted in the nucleoplasm of interphase human cancer cells^2,3^ and Xenopus oocytes^3^. Similarly, many other cytoskeletal proteins were shown to shuttle between the cytoplasm and nucleus, e.g. actin, profilin, α-actinin, plectin and several keratins^4–6^.

Plant tubulin can accumulate in the interphase nucleus during cold treatment^7,8^, from which it is quickly excluded upon re-warming^7^. The quick exclusion of tubulin may be mediated by multiple leucine-rich nuclear export sequences (NESs) found in plant α- and β-tubulin molecules^7^ which are recognized by the Exportin 1/CRM1 receptor of the export pathway. Nuclear export is coupled to the Ras family GTPase Ran and its modulators such as the Ran Guanine Nucleotide Exchange Factor (RanGEF), the Ran-GTPase Activating Protein (RanGAP), and the Ran Binding Proteins 1 and 2 (RanBPs 1/2). The directionality of nuclear transport is proposed to be caused by RanGTP, which binds to and stabilizes the interaction of Exportin 1/CRM1 with its cargo, which in turn greatly facilitates nuclear export (for review see^9–11^). The Ran export pathway was identified in several eukaryotic groups^12–15^ including plants^16^. The mechanism for the accumulation of tubulin into the interphase nucleus is unknown, because a canonical nuclear localization signal (NLS) seems to be absent from both α- and β-tubulins^7,17^. The mechanism and the physiological role of tubulin transport between the nucleus and the cytoplasm in plants is thus poorly understood. In animal cells, nuclear tubulin has been reported in several cultured cell lines^2,18–21^. Tubulin co-precipitated with ASC-2, a transcriptional co-activator amplified in human cancer cells^22^. Further, the βII isoform of beta tubulin, which accumulated in nuclei of cancer cells, could bind to activated Notch1 receptor, modulating Notch1 signaling^23^. Since the Notch signal pathway plays a role in tumorigenesis, the authors suggested that βII isoform in the nucleus may be involved in the regulation of tumor formation. As shown by^2^, soluble tubulin could bind to histone H3. The authors suggested that the role of nuclear tubulin in cancer cell lines was to limit cell proliferation under pathological conditions. To what extent these observations collected from highly abnormal cancer cells can be used to deduce a physiological function for nuclear tubulin, remains an open issue.

In order to get more insight into the molecular aspects of tubulin export, we performed a detailed comparative analysis of tubulin sequences of several organisms. Besides several putative nuclear export sequences already identified in our previous work^7^, additional conserved putative NESs were found in both α- and β-tubulins of distantly related organisms. We tested nuclear export activities for most of these identified putative NESs in plant and animal cultured cells. Our results confirmed that several of the predicted NESs from both α- and β-tubulin were sufficient to drive nuclear exclusion of GFP used as a reporter cargo. Further, based on our observations of nuclear tubulin accumulation in cells treated with leptomycin B, we suggest that the Exportin 1/CRM1 export pathway accounts for the majority of tubulin export from the nucleus, and we discuss the biological significance of tubulin compartmentalization into the cytoplasm during interphase.

## Material and Methods

Identification of nuclear export sequences: tubulin protein sequences from various organisms were manually inspected for the presence of any of following NES consensus sequences: consensus 1: [LIV]-x(1–4)-[LIVFM]-x(2–3)-[LIV]-x-[LIV]^24^, and consensus 2: [LIVFM]-x(2–3)-[LIVFM]-x(2–3)-[LIVFM]-x-[LIVFM]^25^.

### Plant cell culture, transformation and treatment

Cells of tobacco line BY-2 (*Nicotiana tabacum* L. cv Bright-Yellow 2^26^) were cultivated in suspension in darkness at 26 °C on an orbital incubator (120 rpm, orbital diameter 30 mm) in liquid medium (3% [w/v] sucrose, 4.3 g l^−1^ Murashige and Skoog salts, 100 mg l^−1^ inositol, 1 mg l^−1^ thiamin, 0.2 mg l^−1^ 2,4-dichlorophenoxyacetic acid, and 200 mg l^−1^ KH_2_PO_4_ [pH 5.8]) and subcultured weekly (800 ul per 30 ml of medium).

For expression in tobacco cells, all putative nuclear export sequences were cloned into the binary vector *pGreen*^27^ or *pCP60* (kind gift of Prof. Ratet; see Supplementary information 1 for detailed information). The resulting *GFP-NES* vectors were verified by sequencing. For construction of vectors with GFP-tubulin, *GFP-AtTUB6*^28^ gene was used. Restriction sites *XhoI* and *SpeI* were introduced to the N-terminus and C-terminus of the GFP-*AtTUB6* gene, respectively, and these restriction sites were used to clone *GFP-AtTUB6* into the estradiol-inducible *XVE* vector^29^. The resulting vectors were verified by sequencing.

Biolistic transient transformation was performed using 1-μm gold particles coated with vector DNA according to the Helios TM Gene Gun Instruction Manual (Bio-Rad Laboratories, USA) and using 0.05 mg/ml polyvinylpyrrolidone and 1 μg vector DNA per shot. Exponentially growing BY-2 cells (3–5-days after subcultivation) were used for biolistic transformation. Briefly, cells were collected on a filter paper pre-soaked with liquid MS medium and excess of liquid medium was removed by mild suction in Nalgene vacuum filter holder. The cells along with the filter paper were placed on solidified agar-supplemented MS medium in a Petri dish and bombarded immediately using a Helios Gene Gun according to manufacturer’s instructions with a pressure of 150 psi. Cells were observed by fluorescence microscopy 10–16 hours after bombardment. Stable transformation of BY-2 cells was performed according to^30^ with modifications described in^31^. Transgenic cell suspension cultures were maintained as described above, with the addition of 20 μg∙ml^−1^ hygromycin B to the cultivation medium. Expression was induced by the addition of 2 µM β-estradiol into the cultivation medium at least 24 hours before the observation.

### NES and plant tubulin mutagenesis

QuikChange Site-Directed Mutagenesis kit (Stratagene) was used to introduce mutation in selected *GFP-NES* and *AtTUB6* gene in specific amino acid positions of NESβ2 and NESβ3. Manufacturer’s protocol was followed. Briefly, *GFP-AtTUB6* gene and selected *GFP-NES* sequences were cloned into *pGEM* vector. Site-directed mutagenesis was performed using primers listed in the Supplementary information 1. Resulting vectors were confirmed by sequencing. Mutated sequences were cloned into *pGreen* (*GFP-NES*) or *XVE* (*GFP-AtTUB6)* vectors. Mutated *GFP-NES* vectors, designated as *mutNES*, and mutated *GFP-AtTUB6* vectors, designated as *GFP-AtTUB6-mutNES* (for specifications see Supplementary information 1), were expressed in BY-2 cells. Transgenic cell suspension cultures were maintained, and expression of transgenes was induced as described previously.

For Exportin 1/CRM1 inhibitor leptomycin B (Sigma, stock solution 10 μM in methanol treatment, 100 nM concentration is the lowest efficient concentration reported for plant cells^16^ and widely used in nuclear transport studies in plant cell (for example see^32^). Cells expressing selected vectors were incubated with 100 nM leptomycin B for 1 hour.

Transgenic cells were observed by confocal microscopy (LSM 5 Duo, Zeiss, Jena) with filter sets for GFP (excitation at 488 nm, emission at 500–560 nm). Single confocal optical sections through the nuclear region were analysed. At least three independent experiments were conducted for each gene construct. Data represent 10–15 cells and 50 cells per each of the experimental replication of the biolistic transformation and stable transformation, respectively. Representative images are shown.

### Animal cell culture, transfection and treatment

Human U-2 OS (osteosarcoma, ATCC HTB-96) cells were maintained in Dulbecco’s Modified Eagle Medium (DMEM; Gibco) containing 10% fetal bovine serum (Gibco), glutamine and penicillin/streptomycin at 37 °C and 5% CO_2_.

For expression in mammalian cells, all tested NES sequences were cloned into the *pEGFP-C3* vector (Clontech Laboratories, Inc.; Supplementary information 1). The resulting *GFP-NES* vectors were verified by sequencing. Cells grown in glass bottom Petri dishes were transfected using the X-tremeGENE^™^ 9 DNA Transfection Reagent (Sigma) according to the manufacturer’s protocol and observed 36–48 h later. Fluorescence was observed using confocal microscope Zeiss LSM 5 Duo with filter sets for GFP (excitation at 488 nm, emission at 500–560 nm). Single confocal optical sections through the nuclear region were analyzed. At least three independent experiments were conducted for each gene construct. Data represent 20–30 cells per each of the experimental replication. Representative images are presented. Full length human α-tubulin was expressed from the commercially available *pEGFP-Tub* vector (Clontech Laboratories, Inc) as described above.

For leptomycin B treatment, 1 nM concentration is the lowest efficient concentration reported for mammalian cells^33^ and 10 nM concentration is widely used in nuclear transport studies (for example^34^). When indicated, cells were treated with 10 nM leptomycin B (Sigma-Aldrich) by applying the drug into the medium in glass bottom Petri dishes with cells for up to 11 hours.

### Synchronization of BY-2 cells

Tobacco BY-2 cells (stationary cells collected at day 7 after subcultivation diluted tenfold with culture medium) were synchronized using aphidicolin (APC) and propyzamide as described in^35^.

### Analysis of GFP-NES accumulation

GFP-NES accumulation in nuclei of BY-2 and U-2 OS cells was measured as a ratio between the mean gray value in the nucleoplasm and the cytoplasm. The signal intensity was measured using a rectangular selection from ROIs in the nucleus and in the cytoplasm in U-2 OS cells, and using a Freehand selection tool in BY-2 cells. Variants were compared with ANOVA and differences at the level of significance 0.05 in Tukey-HSD post-hoc test were indicated by different letters in graphs.

### Analysis of tubulin accumulation

Images of BY-2 and U-2 OS cells expressing GFP-tubulin were captured using laser scanning confocal microscope Zeiss LSM 5 Duo with the focal plane located in the middle of the nucleus. The mean gray value in the nucleoplasm and the cytoplasm was measured using ImageJ software providing ratio between nuclear and cytoplasmic tubulin content. The signal intensity was measured using a rectangular selection from ROIs in the nucleus and in the cytoplasm in U-2 OS cells, and using a Freehand selection tool in BY-2 cells. Each cell was measured in two different cytoplasmic and nuclear regions and an average value for each cell was used. Statistical significance of differences between mock and leptomycin B-treated cells was calculated using Welch two sample T-test (R statistics software). For each variant and time point, 150 U-2 OS and 50 BY-2 cells were analyzed.

Where indicated, nuclei were labeled using 1 μM *in vivo* nucleic acid stain SYTO 64 (Molecular Probes, 5 mM stock solution in DMSO).

### Protein analysis and tubulin accumulation

U-2 OS cells with stable expression of FUCCI Cell Cycle Sensor^36^ were analyzed on BD Influx cell sorter to obtain samples of 2 × 10^5^ cells double-negative for Cdt1-RFP and geminin-GFP (early G1 phase) and corresponding samples of Cdt1-RFP-positive/geminin-GFP-negative cells (more advanced G1 phase). One set of such samples (referred to as “total samples”) was immediately extracted in sample buffer (15 mM Tris, pH = 6.8, 0.5% SDS, 2.5% glycerol, 1.25% 2-Mercaptoethanol, traces of bromophenol blue) and processed for SDS PAGE/Western blot to allow standardization to total levels of tubulin (using anti-α-Tubulin antibody, cat. no. T9026, Sigma, and Goat Anti-Mouse IgG antibody, 115-035-146, Jackson Immunoresearch). The second set was resuspended in 200 µL cold nuclei extraction buffer (320 mM sucrose, 5 mM MgCl_2_, 10 mM HEPES, 0.25% Triton X-100, pH 7.4), incubated for 10 min on ice, centrifuged at 2000 × g and the pellet was washed twice with phosphate-buffered saline (PBS) to yield clean nuclear samples. Protein samples from nuclei were then extracted and processed as described for total samples. Purity of the nuclear isolation was independently verified using antibodies against cytosolic protein AKT1 (Cell Signaling, #2938) and mitochondrial inner membrane protein SDHA (Abcam, ab14715) as negative controls (data not shown).

## Results

### Potential nuclear export sequences are conserved in all tubulins

**Figure 1:**
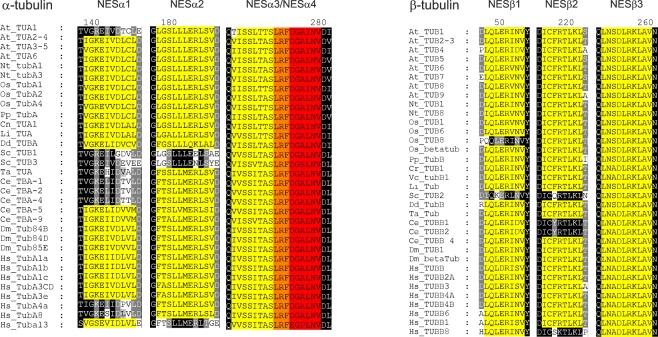
Identification of nuclear export sequences in tubulins. Four putative NESs were found in α-tubulin sequences, designated as NESα1, NESα2, NESα3 and NESα4 (yellow and red color). NESα3 and NESα4 are partially overlapping (orange color). Three putative NESs were found in β-tubulin sequences, designated as NESβ1, NESβ2 and NESβ3 (yellow color). At: *Arabidopsis thaliana*; Nt: *Nicotiana tabacum*; Os: *Oryza sativa*; Cr: *Chlamydomonas reinhardtii*; Li: *Leishmania infantum*; Dd: *Dictyostelium discoideum*; Sc: *Saccharomyces cerevisiae*; Ta: *Trichoplax adhaerens*; Ce: *Caenorhabditis elegan*s; Dm: *Drosophila melanogaster*; Hs: *Homo sapiens*.

The presence of following NES consensus sequences was screened: consensus 1 ([LIV]-x(1–4)-[LIVFM]-x(2–3)-[LIV]-x-[LIV])^24^, and consensus 2 ([LIVFM]-x(2–3)-[LIVFM]-x(2–3)-[LIVFM]-x-[LIVFM])^25^. Four putative NESs were found in α-tubulins, and three in β-tubulin sequences of *Arabidopsis thaliana*. They were designated as NESα1–4 for α-tubulins and NESβ1–3 for β-tubulins (Fig. 1). We investigated α- and β- tubulins of various organisms for the presence of these putative nuclear export sequences. The analysis showed that the nuclear export sequences identified in *Arabidopsis* tubulins were rather conserved also in tubulins of the other organisms studied, including human tubulins (Fig. 1). The most conserved putative NESs were NESα3, NESα4 and NESβ3. The least conserved was NESα1, which was found to vary even among the isoforms of tubulin in *Arabidopsis* and human genome. *Saccharomyces cerevisiae* is the most diverged organism from those included in our analysis, and contains only NESα3, NESα4 and NESβ3 motifs (Fig. 1). Interestingly, NESα3 and NESα4 sequences partially overlap.

Generally, all organisms compared harbor at least one putative NES sequence in conserved regions of their tubulins.

### Putative tubulin NESs confer nuclear export activity in plant and animal cell model

**Figure 2:**
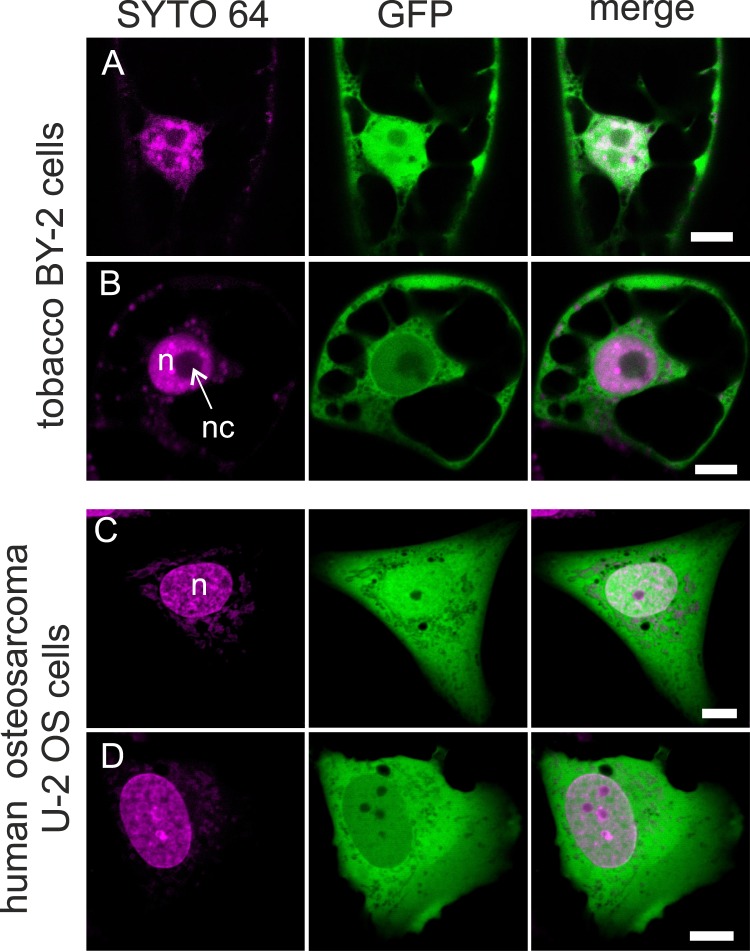
GFP localization in model cells. Free GFP (green) expressed in tobacco BY-2 cells (**A**) and human osteosarcoma U-2 OS cells (**C**) is localized in the cytoplasm and in the nucleus (magenta), where it is often accumulated. Concentration of GFP fused to a functional nuclear export sequence (NESα2 from TUA3) is clearly reduced in the nucleus when compared to cytoplasm in BY-2 (**B**) and U-2 OS (**D**) cells. One optical section of a confocal microscope through nuclear region is shown. Nucleus (n), nucleolus (nc). Scale bar: 10 µm.

To verify functionality of the predicted tubulin nuclear export sequences out of the tubulin molecule context, we fused the respective putative nuclear export sequence to the C-terminus of GFP. The nuclear export activity of fusion proteins was then tested in the model plant cell line BY-2 (Bright Yellow-2, tobacco cell line^26^), and in the model mammalian cell line U-2 OS (osteosarcoma human cell line, ATCC HTB-96, well suited for live-cell imaging owing to its flat morphology with big nuclei). Free GFP was used as a negative control homogenously distributed to both the cytoplasm and the nucleoplasm in tobacco and U-2 OS cells (Fig. 2A,C). The intensity of the free GFP signal was comparable between the cytoplasm and the nucleoplasm in U-2 OS, while in tobacco cells, the signal accumulated in nuclei. Thus, the criterion for a validated activity as NES was a significant decrease of signal in the nucleoplasm, accompanied by an increase of signal in the cytoplasm (Fig. 2B,D). Using this criterion, BY-2 and U-2 OS cells transiently expressing the tested GFP-NES fusions were inspected under the confocal microscope and the number of cells exporting GFP from the nucleus was assessed.

**Figure 3:**
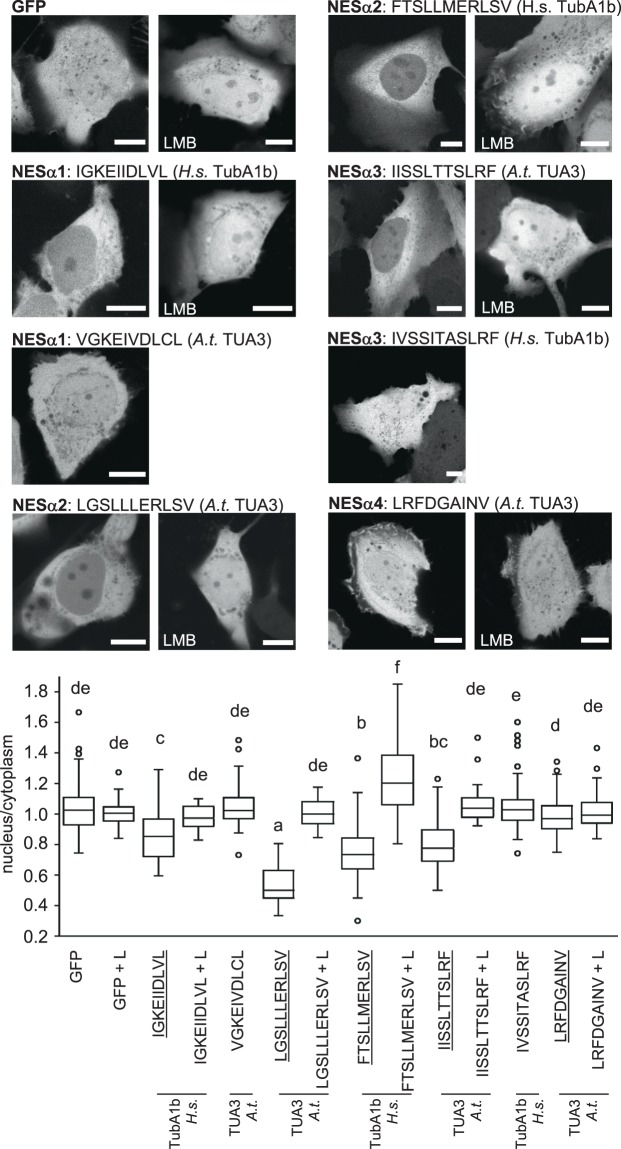
Quantification of export capacity of NESs from *A. thaliana* and *H. sapiens* α-tubulins fused to GFP and expressed in human osteosarcoma U-2 OS cells. Examples of cells expressing the respective GFP-NES construct are shown. Quantification confirmed export activity of Arabidopsis NESα2, NESα3 and NESα4, and human NESα1 and NESα2. Export activity was not confirmed for Arabidopsis NESα1 and human NESα3. All exporting NESs were sensitive to leptomycin B treatment (LMB). Free GFP was the control. Exporting NESs are underlined. Variants were compared with ANOVA and differences at the level of significance 0.05 in Tukey-HSD post-hoc test were indicated by different letters in graphs. L, LMB: leptomycin B. Scale bar = 10 μm.

**Figure 4:**
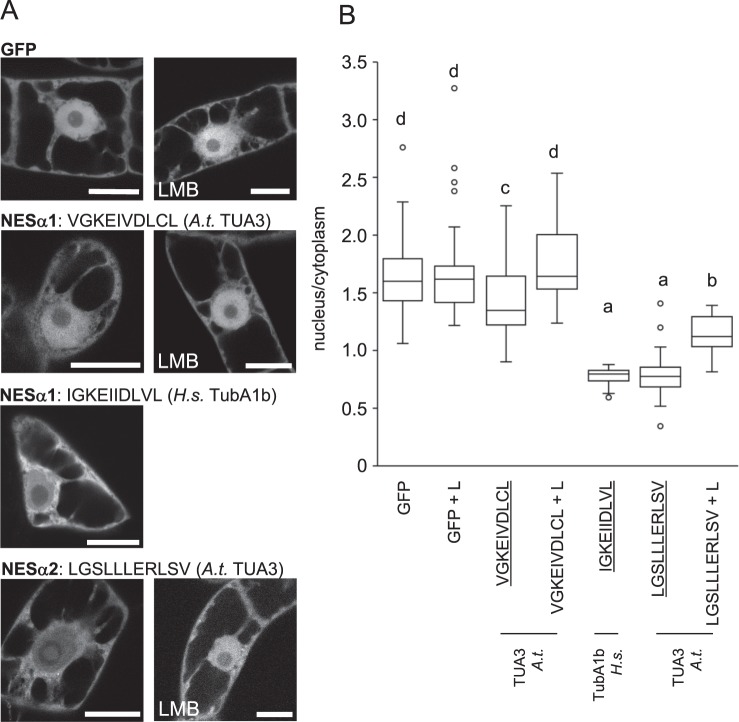
Quantification of export capacity of NESs from *A. thaliana* and *H. sapiens* α-tubulins, fused to GFP and expressed in tobacco BY-2 cells. (**A**) Examples of cells expressing respective GFP-NES construct are shown. (**B**) Quantification confirmed export activity of Arabidopsis NESα1 and NESα2, and human NESα1. Most exporting NESs were sensitive to leptomycin B treatment (LMB). Free GFP was the control. Exporting NESs are underlined. Variants were compared with ANOVA and differences at the level of significance 0.05 in Tukey-HSD post-hoc test were indicated by different letters in graphs. L, LMB: leptomycin B. Scale bar = 20 μm.

**Figure 5:**
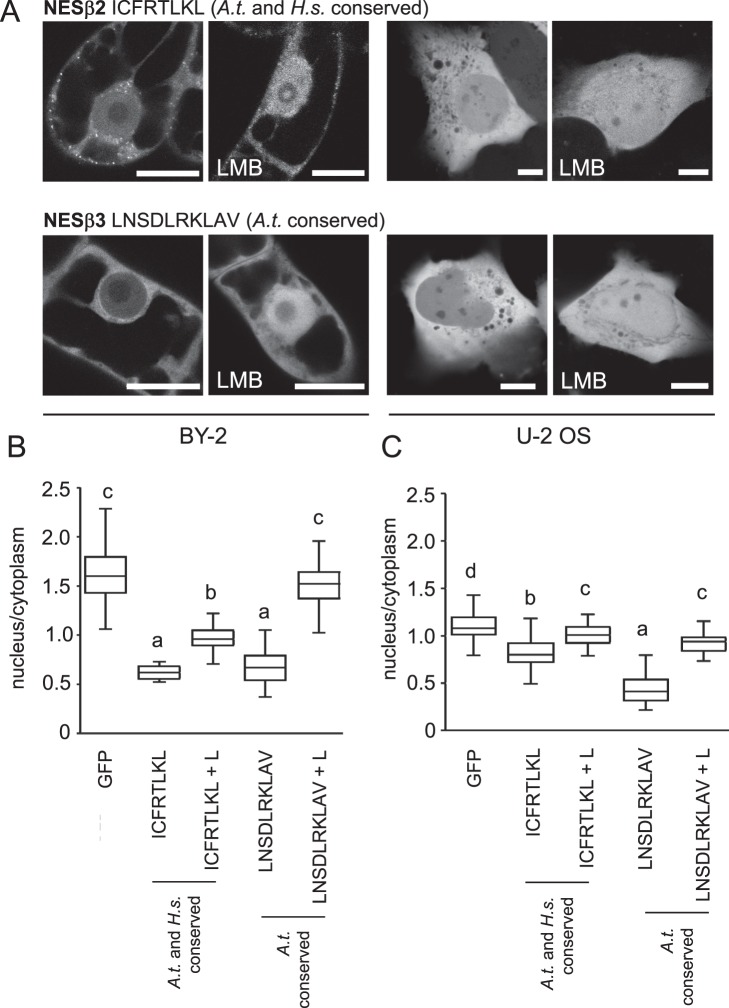
Quantification of export capacity of NESs from *A. thaliana* and *H. sapiens* β-tubulins, fused to GFP and expressed in tobacco BY-2 and human U-2 OS cells. (**A**) Examples of cells expressing respective GFP-NES construct are shown. (**B,C**) Quantification confirmed export activity of both NESβ2 and NESβ3 in BY-2 (**B**) and U-2 OS (**C**) cells. Both NESs were sensitive to leptomycin B treatment. Variants were compared with ANOVA and differences at the level of significance 0.05 in Tukey-HSD post-hoc test were indicated by different letters in graphs. L, LMB: leptomycin B. Scale bar = 20 μm (BY-2), 10 μm (U-2 OS).

Putative nuclear export signals from both *Arabidopsis* and human tubulins were tested in both experimental systems to learn more about their conserved functions. In the first experiment, vectors were expressed transiently and frequency of cells showing nuclear export was scored (Table 1). NESs which were never observed to export GFP in any of biological repetitions were considered inactive in our export assay and were not pursued further, while NESs showing export of GFP from the nucleus were analysed further. Quantification was performed for exporting vectors of U-2 OS cells (Figs 3 and 5). For quantification in tobacco cells, vectors were expressed stably in BY-2 cells, which allowed quantification of export activity in large cell population (Figs 4 and 5). The activity of NESs exporting GFP was further verified by leptomycin B inhibition of their nuclear export activity (Figs 3 and 4).

**Table 1:** Putative tubulin NESs were tested for their capacity to export GFP from nuclei of tobacco BY-2 and human U-2 OS transiently transformed cells.

AA sequence	NES	Tubulin isoform	Percetage of exporting tobacco cells	Percetage of exporting human cells
VGKEIVDLCL	NESα1	*A.t*. TUA3	5.6	37.3
IGKEIIDLVL	NESα1	*H.s*. TubA1b	100	79.6
LGSLLLERLSV	NESα2	*A.t*. TUA3	89.8	100
FTSLLMERLSV	NESα2	*H.s*. TubA1b	0	93
IISSLTTSLRF	NESα3	*A.t*. TUA3	0	97.5
IVSSITASLRF	NESα3	*H.s*. TubA1b	0	40.9
LRFDGAINV	NESα4	*A.t*. TUA3	0	15.2
LQLERINV	NESβ1	*A.t*. and *H.s*.	0	0
ICFRTLKL	NESβ2	*A.t*. and *H.s*.	64.1	92.2
LNSDLRKLAV	NESβ3	*A.t*. conserved	55.2	98.8

Number of cells showing export of GFP is expressed as percentage of analysed cells. At least three independent experiments were conducted for each gene construct. Approximately 40 cells and 80 cells were analysed in total for tobacco and human cells, respectively.

Quantification of nuclear export activity confirmed export activity of most sequences with the exception of NESα1 from TUA3 (VGKEIVDLCL) and NESα3 from TubA1b (IVSSITASLRF) in U-2 OS cells (Fig. 3). Functionality of some NESs from α-tubulins was partially conserved over tobacco and human cells, while activity of some NESs seemed to be confined to one model system only. For instance, NESα1 from *Arabidopsis* tubulin TUA3 (VGKEIVDLCL) showed rather weak export activity in tobacco cells and failed to export in human cells, while NESα1 from human TubA1b (IGKEIIDLVL) had clear export activity in both types of cells (Figs 3 and 4). In contrast to these motifs, the NESα2 motif from *Arabidopsis* TUA3 (LGSLLLERLSV) exported strongly in tobacco and human cells (Figs 3 and 4), but NESα2 from human tubulin TubA1b (FTSLLMERLSV) showed nuclear export activity in human cells only (Fig. 3, Table 1). There were also cases of inversed activity: neither NESα3 from *Arabidopsis* (IISSLTTSLRF), nor human tubulins (IVSSITASLRF) did export GFP from nuclei of tobacco cells (see Table 1), but Arabidopsis NESα3 showed export activity in U-2 OS human cells (Fig. 3). A conserved NESα4 did not export GFP from tobacco nuclei, and only weakly exported in human cells (Fig. 3, Table 1).

In contrast to their α-tubulin counterparts, putative NESs from β-tubulins showed similar export properties in both models. NESβ1 exported GFP neither from nuclei of tobacco nor from human cells (Table 1), while NESβ2 and NESβ3 showed strong and leptomycin B-sensitive GFP export from nuclei of both tobacco and human cells (Fig. 5).

In summary, all putative α-tubulin motifs showed nuclear export activity in at least one model. NESα1 from human TubA1b and NESα2 from *Arabidopsis* TUA3 were found to be strong export signals in our export tests. NESα4 was the weakest NES tested, nevertheless, it was sensitive to leptomycin B (Fig. 3). In β-tubulin, NESβ1 was inactive in nuclear export. In contrast, NESβ2 and NESβ3 efficiently conferred export in our tests.

### Mutagenesis of tubulin NESs

**Figure 6:**
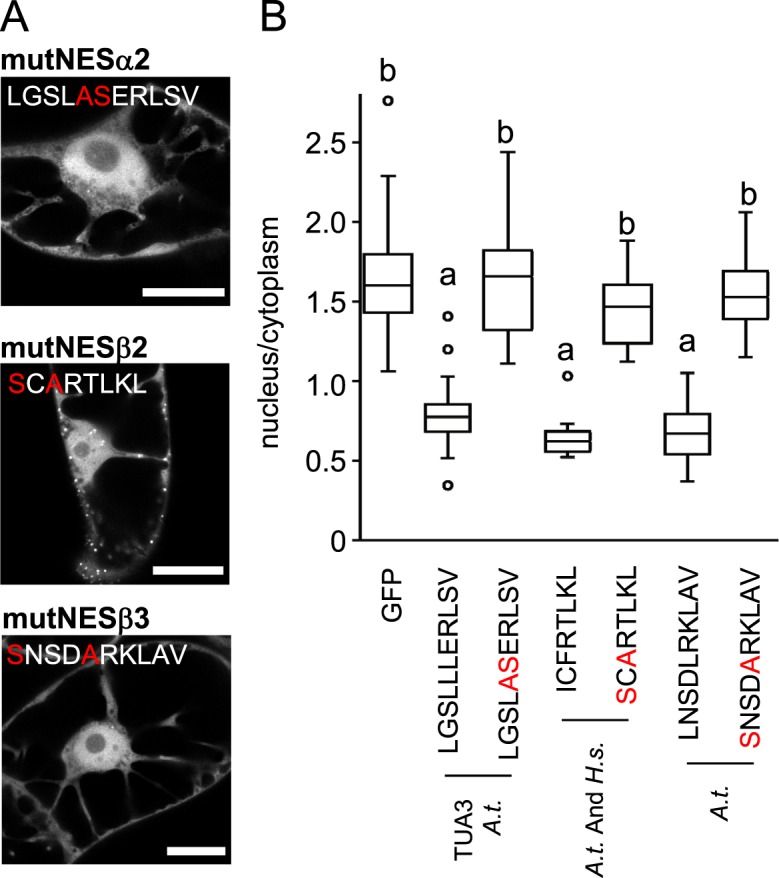
Mutagenesis of NES inhibits the export activity on tobacco cells. (**A**) Introduction of amino acid substitution into sequences of GFP-NESα2 (mutNESα2), GFP-NESβ2 (mutNESβ2) and GFP-NESβ3 (mutNESβ3) from *A. thaliana* tubulins (indicated in red), which resulted in absence of any known NES consensus sequences, prevents the export of GFP from the nucleus in BY-2 cells. (**B**) Quantification confirmed that nucleus/cytoplasmic ratio of mutated sequences was similar to GFP, thus losing its export capacity. Variants were compared with ANOVA and differences at the level of significance 0.05 in Tukey-HSD post-hoc test were indicated by different letters in graphs. Scale bar = 20 μM.

In order to test the sensitivity of export signals to sequence changes, we introduced point mutations in selected GFP-NES sequences that either preserved or disrupted consensus of a given nuclear export sequence. Figure 6 shows that introduction of a mutation breaking the consensus resulted in the inhibition of nuclear export activity of NESα2 (mutNESα2), NESβ2 (mutNESβ2) and NESβ3 (mutNESβ3).

Introduction of point mutation mutNESβ2 or mutNESβ3 in full-length GFP-β-tubulin molecule fused to GFP resulted in dramatic loss of protein incorporation into microtubules (Supplementary information 2). GFP-AtTUB6-mutNESβ2 was found to assemble into sparse polymers or tubulin clusters (Supplementary information 2C-F); mutNESβ3 β-tubulin was never observed to form microtubules and always showed diffuse cytoplasmic localization (Supplementary information 2 G, H).

### Export activity conferred by tubulin NESs is inhibited by leptomycin B

We probed for the effect of Exportin 1/CRM1 inhibitor leptomycin B^37,38^ on the export activity of most active export signals expressed in BY-2 and U-2 OS cells. As shown in Figs 3–5, treatment with leptomycin B effectively inhibited GFP export from the nucleus for tested signals in both types of cells, which suggests their conserved interaction with the Exportin 1/CRM1 pathway. Leptomycin B treatment did not change nuclear:cytoplasmic signal ratio in cells expressing free GFP in U-2 OS (Fig. 3) and BY-2 (Fig. 4) cells.

### Tubulin accumulates in nuclei of cells treated with leptomycin B

**Figure 7:**
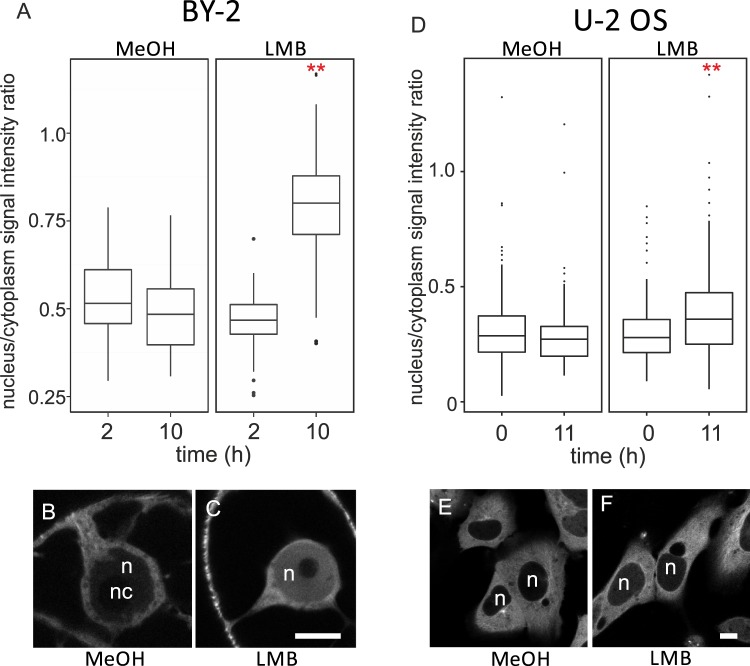
Prolonged exposure to leptomycin B (LMB) induces accumulation of tubulin in nuclei. BY-2 and U-2 OS cells expressing GFP-tubulin were treated with 100 nM and 10 nM LMB, respectively, and the amount of tubulin in cytoplasm and nucleus was evaluated. Whereas the amount of tubulin in nuclei of both BY-2 (**A**) and U-2 OS (**D**) cells was comparable to mock (methanol, MeOH) - treated cells, prolonged exposure to LMB resulted in significant increase of nuclear tubulin signal (**A,D**) in BY-2 cells (**B,C**) and U-2 OS cells (**E,F**). Statistics: Welch Two Sample t-test; p-value = 0.066 (MeOH in A), p-value < 0.001 (LMB in A); p-value = 0.151 (MeOH in D), p-value < 0.001 (LMB in D). Nucleus (n), nucleolus (nc). Scale bar = 10 μM.

Sensitivity of nuclear export to leptomycin B suggests that tubulin actively shuttles between cytoplasm and nucleus in eukaryotic cells, and that this process depends on the Exportin 1/CRM1 pathway. We therefore asked, whether tubulin accumulates in nuclei in response to treatment with leptomycin B as inhibitor of the Exportin 1/CRM1 pathway. In fact, we observed that the treatment of GFP-tubulin expressing BY-2 and U-2 OS cells resulted in accumulation of tubulin in nuclei. The accumulation was measured as a ratio between nuclear and cytoplasmic signal of GFP-tubulin in interphase nuclei. While after 2 hours of treatment with 100 nM leptomycin B treatment, nuclear GFP-tubulin signals were comparable to mock-treated cells in BY-2 cells, the nuclear signal had significantly increased after 10 hours (Fig. 7A–C). Likewise, for U-2 OS cells, treatment with 10 nM of leptomycin B produced a significant increase of the nuclear signal within 11 hours, although the response was less conspicuous as compared to BY-2 cells (Fig. 7D–F).

**Figure 8:**
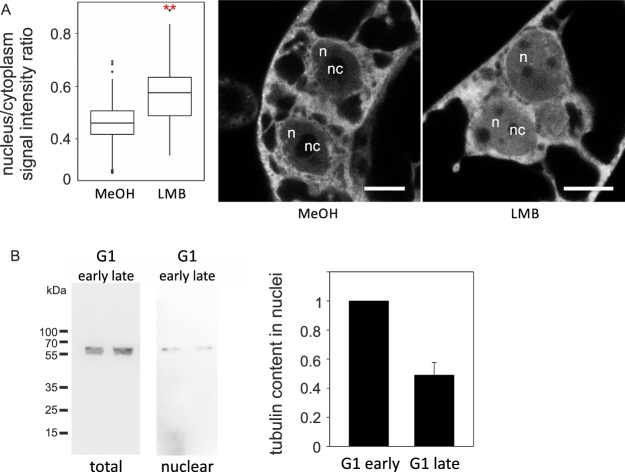
(**A**) Short LMB treatment (2 hours) is sufficient to induce tubulin accumulation in BY-2 cells entering G1 phase. Synchronized BY-2 cells expressing GFP-tubulin were treated with 100 nM LMB at the moment of telophase peak and cultivated for another 2 hours allowing cells to reach early G1 phase. Early G1 cells were evaluated for GFP-tubulin content. LMB-treated cells contained significantly more tubulin in the nucleus when compared to mock (methanol, MeOH) - treated cells. Statistics: Welch Two Sample t-test, p-value < 0.001. Nucleus (n), nucleolus (nc). Scale bar = 10 μM. (**B**) Tubulin content decreases during the course of G1 phase. Populations of FUCCI U-2 OS cells in early and late G1 phase were sorted. Tubulin content was analyzed in total lysate and nuclear fraction of cells in early and late G1 phase using anti-α-tubulin antibody. Early G1 phase nuclei contain more tubulin relative to the tubulin content in nuclei of late G1 phase. Data from 3 independent experiments. Error bar = SE.

These results are consistent with the hypothesis that tubulin is removed from the nucleus by Exportin 1/CRM1 pathway. Prolonged exposure to leptomycin B was needed to obtain significant tubulin accumulation in nuclei, which indicates that tubulin enters nuclei slowly. To test, whether the amplitude of the intranuclear signal depends on mitotic activity and the nuclear envelope integrity, we followed the nuclear tubulin accumulation in BY-2 cells that were synchronized and treated with leptomycin B at the time of the mitotic peak. The intranuclear content of GFP-tubulin was then quantified in early G1 nuclei, 2 hours after the leptomycin B addition. As shown in Fig. 8A, under these conditions, a significant increase of GFP-tubulin signal was detected as early as 2 hours after addition of this export inhibitor. Thus, the response to leptomycin B became much faster in cells undergoing mitosis.

This has led us to investigate further temporal dynamics of nuclear tubulin after successful mitosis. We therefore employed an U-2 OS cell line stably expressing the FUCCI Cell Cycle sensor with fluorescently tagged cell cycle oscillators Cdt1and geminin as markers. Cells start to accumulate Cdt1-RFP during the course of the G1 phase and later degrade it at the entry into the S-phase. Geminin-GFP accumulates at the onset of the S-phase and degrades at the entry into mitosis, creating a short window of double-negative cells in the very early G1-phase^39^. We used cell sorting to obtain clear populations of G1-early and G1-late cells and characterised their total tubulin content as well as amount of tubulin in the nucleus. While the total levels of tubulin remained approximately unchanged between early and advanced G1 phase, the abundance of nuclear tubulin in FUCCI cells decreased during the progression through G1 phase as advanced G1-phase cells had approximately 50% less tubulin in nuclei than early G1 cells (Fig. 8B).

## Discussion

In this work, we showed that tubulins from both, plants and animals, harbor highly conserved sequences with nuclear export activity. These nuclear export signals were able to confer nuclear export activity to GFP as reporter cargo, and some of these signals are functionally conserved between animal and plants. The export activity can be inhibited by leptomycin B, indicative of the Exportin 1/CRM1 pathway.

Most nuclear export sequences identified in our screen were highly conserved across various species. This may be attributable to the mere fact that tubulin is generally very highly conserved. Indeed, the most conserved nuclear export sequences such as NESβ3, which are present in all studied species, are found in highly conserved regions of tubulins. The importance of these conserved regions for tubulin assembly is further documented by our observation that introduction of point mutations into NESβ2 and NESβ3 region of the complete β-tubulin interferes with its assembly into microtubules. The highest divergence from the typical set of NESs was identified in *S. cerevisiae*. However, we noticed that all tested species (including *S.c*.) contained at least one functional NES in each, α- and β-tubulin. This corroborates the importance of nuclear export signals in tubulins. Interestingly, NESβ3 localizes to a domain involved in interactions between α- and β-tubulin subunits (Supplementary information 3). Since β-tubulin does not exist as monomer, it remains to be elucidated, whether this conserved signal is sufficiently exposed to interact with Exportin 1/CRM1.

When free GFP is expressed, it localizes to both, cytoplasm and karyoplasm, because it can freely diffuse through the nuclear pores, since monomeric GFP molecule is smaller than the size exclusion of the pore complex^40^. Although GFP lacks canonical NLS^40,41^, it is more abundant in the nucleus in most cases, which might be linked with to lower mobility in and from the karyoplasm due to generally higher karyoplasm viscosity. When fused to an active export sequence, GFP preferentially localized to the cytoplasm. Since such localization was never observed in cells expressing free GFP, all sequences capable of preferential cytoplasmic localization of GFP were obviously able to confer export to their cargo. This operational criterion for active nuclear export sequences holds true, independently of the question, whether free GFP is preferentially seen in the nucleus (as in tobacco BY-2), or whether it is equally distributed between nucleus and cytoplasm (as in mammalian U-2 OS cells).

We expressed all putative NESs transiently in order to select for active sequences. Export activity of NESs showing active export were then quantified in U-2 OS transfected cells and stably transformed tobacco cells. We observed that the export functionality and efficiency was strongly dependent on the type of NES. For example, *Arabidopsis* NESα1 (VGKEIVDLCL) exported GFP only in tobacco cells, but quantification in human cells did not reveal statistically significant export function. In contrast, human NESα1 (IGKEIIDLVL) exported GFP in a both tobacco and human cells. A very strong export activity in both, tobacco and human, cells was confirmed for Arabidopsis NESα2 (LGSLLLERLSV), and conserved NESβ2 (ICFRTLKL) and NESβ3 (LNSDLRKLAV). Our observations are consistent with the published record that NES sequences identified so far are very diverse and their affinity to Exportin 1/CRM1 may differ^42^. To further validate and dissect the molecular function of the tubulin NESs, the interaction between tubulin and Exportin 1/CRM1 should be addressed biochemically using approaches such as pull-downs of native and mutated versions of tubulin. Such approaches are also needed to resolve the activity of NESβ3, which is not exposed to the heterodimer surface, or of NESβ1, which we found to be inactive but was considered active by other authors^2^. The latter phenomenon is interesting also in the light of our finding that not only β-tubulins, but also all α-tubulins contain active NESs recognized by Exportin1/CRM1 pathway.

Our results suggest that α- and β-tubulins contain multiple active NESs that mediate tubulin export from the nucleus. How tubulin can enter the nucleus, has remained elusive. None of the canonical nuclear localization signals (NLS) is detectable in tubulin sequences^7,17^. It is unlikely that tubulin enters the nucleus passively, since the tubulin heterodimer has a molecular weight of 110 kDa, extended to 138 kDa by the GFP tag, which is far above the size exclusion of nuclear pores. Tubulin might be imported in a complex with unknown proteins containing an active NLS. However, given the fact that different types of NLS have been found correlated with a considerable diversity in the molecular mechanism of uptake (reviewed in^43^), the possibility of direct import through different, still unknown mechanisms cannot be ruled out.

Our experiments indicate a third mechanism underlying nuclear accumulation of tubulin. While the accumulation of GFP-tagged tubulin after treatment with leptomycin B required several hours to become significant in non-synchronized tobacco BY-2 cells, it was accelerated considerably, when cells were synchronized, such that significant accumulation was evident already two hours after the inhibitor addition. This phenomenon could be explained by a scenario, where tubulin approaches the chromatin during mitosis, when nuclear envelope is disassembled and forms no barrier to cytosolic proteins, and is subsequently trapped in the nucleus at the end of mitosis by the newly assembled nuclear envelope. This is also corroborated by our finding that tubulin levels decrease during the course of the G1 phase. This trapping hypothesis is consistent with results of^19^, who investigated the mechanism of α/β_II_ tubulin heterodimer localization in the nucleus of cultured rat kidney mesangial cells. Here, microinjected tubulin was present in nuclei only in cells that underwent mitosis, suggesting that tubulin was captured in the nucleus during nuclear envelope reassembly at the end of the mitosis.

This tubulin-trapping model does not exclude real nuclear import of tubulin through nuclear pores. In fact, two arguments indicate that tubulin can also enter the nucleus during interphase: The significant increase to around twice the level seen after 10 hours of leptomycin B treatment in non-synchronized tobacco BY-2 cells (when compared to untreated cells) cannot be fully attributed to mitotic trapping, since only around 5% of these cells undergo mitosis. Furthermore, the relative amount of tubulin in early G1 nuclei of synchronized cells (resulting from trapping) was smaller than in nuclei of non-synchronized cells after 10 hours of leptomycin B treatment, indicative of additional tubulin entering during period between early G1 and the next G2/M transition. In the U-2 OS cells, the nuclear tubulin accumulation in response to leptomycin B is much weaker. This might mean that real tubulin import through the nuclear envelope does not play a role in the animal model, while it does play a certain (although not dominant) role in the plant model. Indeed, transgenic tobacco lines expressing tubulin fused to GFP show a weak nuclear signal (Supplementary information 2J). However, tubulin was never observed to form microtubules in nuclei of U-2 OS and BY-2 cells (Supplementary information 2I and 2J, respectively).

The finding of active tubulin export from the nucleus that is conserved over organisms as different as *Homo sapiens* and *Arabidopsis thaliana* indicates that the exclusion of tubulin from the karyoplasm must be vital. Since proteins involved in tubulin nucleation and microtubule organization during mitosis are found in the nucleus^44^, the tubulin exclusion might have the function to suppress precocious initiation of mitotic events. In fact, several nuclear proteins can interact with α- and β-tubulin. This not only includes γ-tubulin, a molecule involved in the nucleation of microtubules, which is present in nuclei of plant^45^ and mammalian cells^46,47^, but also plant TPX2, a microtubule-binding protein that remains nuclear during interphase, but is exported from the nucleus at the G2/M phase transition to regulate the spindle assembly^32^. Intriguingly, a typical nuclear protein involved in DNA organization, histone H1, was shown to nucleate microtubules at the nuclear rim of tobacco cells^48^. All these examples suggest that some proteins with microtubule-nucleating activity reside in interphase nucleus and assist the organization of microtubules during mitosis, when the nuclear envelope disassembles. It is therefore plausible that the strict compartmentalization of tubulin to the cytoplasm during interphase prevents illegitimate interaction with microtubule-nucleating factors. Cancer cell lines were repeatedly reported to contain nuclear tubulin^18–22,49^, which suggests that cell cycle control-related defects are associated with the loss of tubulin localization control. We also cannot exclude a possibility that tubulin shuttling between the nucleus and the cytoplasm is required for an unknown function of tubulin within interphase nuclei. It is possible that low concentrations of tubulin, which are below the critical concentration needed for tubulin polymerization, play a regulatory role, as was shown for actin molecules^50^. The recent finding that a non-canonical class-XIV kinesin, dual localization kinesin, can enter the nucleus of tobacco BY-2 cells in response to cold stress, which is followed by the specific activation of cold box factor 4, a central switch for cold hardening^51^ would support such a regulatory function also for plant cells.

### Summary

Here we show the functionality of multiple nuclear export sequences, which are conserved between different eukaryotic kingdoms. Their function can be disrupted by leptomycin B, indicative of interaction with the Exportin 1/CRM1 pathway. These sequences enable the export of tubulin from nuclei. We propose that this system enables exclusion of tubulin trapped in interphase nuclei during re-establishing of nuclear envelope after the mitosis (acting in concert with active tubulin entry during the interphase, at least in plant cells). The main biological function of tubulin exclusion from the interphase nucleus might be the suppression of mitotic structures prior to the G2/M transition.

## Supplementary information

Supplementary information
