# Supplementary material for: Tubulin is actively exported from the nucleus through the Exportin1/CRM1 pathway
Source: Sci Rep. 2019 Apr 5;9:5725. doi: 10.1038/s41598-019-42056-6 (PMC6451007; doi:10.1038/s41598-019-42056-6)

Supplementary Information

Tubulin is actively exported from the nucleus through the Exportin1/CRM1 pathway

Schwarzerová K[1*], Bellinvia E[1], Martinek J[1], Sikorová L[1], Dostál V[2], Libusová L[2], Bokvaj P[1], Fischer L[1], Schmit A.C.[3], Nick P[4]

[1]Department of Experimental Plant Biology, Faculty of Science, Charles University, Viničná 5, Prague, Czech Republic

[2]Department of Cell Biology, Faculty of Science, Charles University, Prague, Viničná 7, Czech Republic

[3]Institut de Biologie Moléculaire des Plantes, Centre National de La Recherche Scientifique, Université de Strasbourg, F67084, Strasbourg-cedex, France

[4]Molecular Cell Biology, Botanical Institute, Karlsruhe Institute of Technology (KIT), Fritz-Haber-Weg 4, 76131, Karlsruhe, Germany

*corresponding author

Corresponding author e-mail: schwarze@natur.cuni.cz

Supplementary information 1:

a) NES oligonucleotides
b) Mutated NES oligonucleotides for site-directed mutagenesis of GFP-NES
c) Oligonucleotides for β-tubulin site-directed mutagenesis
d) Vector map of *pCP60*

Oligonucleotides corresponding to NES sequences in different tubulin isoforms of *A. thaliana* and *H. sapiens* were designed and cloned into *pCP60* or *pGreen* [28], and into *pEGFP-C3* vector (Clontech Laboratories, Inc), respectively, to create *GFP-NES* expression vectors. Origin of each sequence, type of expression vector with specified restriction sites and target species are also indicated. Please note that 0029 and 0129 *pGreen* vectors were engineered to include 35S promotor and pA terminator sequences.

Supplementary information 2:

Tobacco cell line BY-2 stably transformed with full-length GFP-AtTUB6 carrying mutation in NESβ2 and NESβ3 position observed using confocal scanning microscope. GFP-AtTUB6 protein is incorporated into microtubules (A, one central optical section). GFP-AtTUB6-mutNESβ2 (C) and GFP-AtTUB6-mutNESβ3 (G) proteins are not incorporated into microtubules. Central optical sections are shown. (B, D-F, H) Analysis of cortical microtubules assembly status using VAEM observation method on TIRF-equipped microscope. (B) GFP-AtTUB6 forms numerous cortical microtubules. (D-F) GFP-AtTUB6-mutNESβ2 expressing cells do not show GFP-labelled microtubules (D) or contain only fragmented sparse polymers (E, F), suggesting poor incorporation into microtubules. (H) No cortical microtubules were observed in cells expressing GFP-AtTUB6-mutNESβ3 expressing cells. Scale bar = 10 μm.

(I) Confocal image of U-2 OS cell stained using anti-tubulin antibody. Central optical section through the middle portion of a cell nucleus, deconvolved using Huygens software to improve resolution in Z. (J) Confocal image of BY-2 cells expressing GFP-AtTUB6. Central optical section.

Supplementary information 3:

Localization of NESs in the 3D structure of the tubulin heterodimer. 3D models were generated in RCSB PDB Protein Workshop 4.2.0 (Moreland et al. 2005) using crystal structure of tubulin heterodimer from *Sus scrofa* (1TUB, Nogales et al. 1998). α-tubulin subunit is highlighted in yellow, β-tubulin subunit in green. NESs are highlighted in red. A - NESα1 (IGKEIIDLVL), NESα2 (FTSLLMERLSV), NESα3 (VSSITASLRF), NESα4 (LRFDGALNV); B - NESβ1 (LQLERINV), NESβ2 (ICFRTLKL), NESβ3 (LNADLRKLAV).

References:
Moreland JL, Gramada A, Buzko OV, Zhang Q, Boume PE (2005) Molecular Biology Toolkit (mbt): A Modular Platform for Developing Molecular Visualization Applications. BMC Bioinformatics 6:21
Nogales E, Wolf SG, Downing KH (1998) Structure of the alpha beta tubulin dimer by electron crystallography. Nature 391: 199-203

Supplementary information 1

a) NES sequences

Labeled as	Protein	AA sequence	Forward oligo	Reverse oligo	Sequence origin	Restriction sites	Expression vectors	For expression in:
NESα1	TubA1a,TubA1b,TubA1c	IGKEIIDLVL	*ATGAGTAAAGGAGAAGAAAC*	*TTACAACACAAGGTCAATGATCTCCTTGCCAATGTATTTGTATAGTTCATCCATG*	H.s. alpha	*BamHI - HindIII*	*0029 pGreen*	N.t. BY-2
NESα1	TubA1a,TubA1b,TubA1c	IGKEIIDLVL	*GTACCCCGGGATTGGCAAGGAGATCATTGACCTCGTGTTGTAG*	*GATCCTACAACACGAGGTCAATGATCTCCTTGCCAATCCCGGG*	H.s. alpha	*BamHI - BsrGI*	*pEGFP-C3*	H.s. U-2 OS
NESα1	TUA3-5	VGKEIVDLCL	*GATCCAGTTGGGAAGGAAATTGTGGATCTATGTCTTTAGGAGCT*	*CCTAAAGACATAGATCCACAATTTCCTTCCCAACTG*	A.t. alpha	*BamHI - SacI*	*pCP60*	N.t. BY-2
NESα1	TUA3-5	VGKEIVDLCL	*GTACCCCGGGGTTGGTAAAGAAATCGTAGACCTTTGTCTTTAG*	*GATCCTAAAGACAAAGGTCTACGATTTCTTTACCAACCCCGGG*	A.t. alpha	*BamHI - BsrGI*	*pEGFP-C3*	H.s. U-2 OS
NESα1	TUA1	VGREIVDTCL	*GATCCAGTTGGAAGGGAAATTGTTGACACCTGCCTTTAG*	*AGCTTCTAAAGGCAGGTGTCAACAATTTCCCTTCCAACTG*	A.t. alpha	*BamHI - HindIII*	*0029 pGreen*	N.t. BY-2
NESα1	TUA1	VGREIVDTCL	*GTACCCCGGGGTTGGAAGGGAAATTGTTGACACCTGCCTGTAG*	*GATCCTACAGGCAGGTGTCAACAATTTCCCTTCCAACCCCGGG*	A.t. alpha	*BamHI - BsrGI*	*pEGFP-C3*	H.s. U-2 OS
NESα2	TubA1a,b,c, TubA4a	FTSLLMERLSV	*GATCCATTTACTTCTCTTCTTATGGAAAGACTTTCTGTTTAGGAGCT*	*CCTAAACAGAAAGTCTTTCCATAAGAAGAGAAGTAAATG*	H.s. alpha	*BamHI - SacI*	*pCP60*	N.t. BY-2
NESα2	TubA1a,b,c, TubA4a	FTSLLMERLSV	*GTACCCCGGGTTCACCTCCCTGCTCATGGAACGGCTCTCCGTTTAG*	*GATCCTAAACGGAGAGCCGTTCCATGAGCAGGGAGGTGAACCCGGG*	H.s. alpha	*BamHI - BsrGI*	*pEGFP-C3*	H.s. U-2 OS
NESα2	TUA1-6	LGSLLLERLSV	*ATGAGTAAAGGAGAAGAAAC*	*TTATACGGACAAGCGCTCCAAACAGTAAAGAACCCAAGTATTTGTATAGTTCATC*	A.t. alpha	*XbaI - BamHI*	*pCP60*	N.t. BY-2
NESα2	TUA1-6	LGSLLLERLSV	*GTACCCCGGGTTGGGTTCTCTGTTGCTAGAGCGTTTGTCTGTATAG*	*GATCCTATACAGACAAACGCTCTAGCAACAGAGAACCCAACCCGGG*	A.t. alpha	*BamHI - BsrGI*	*pEGFP-C3*	H.s. U-2 OS
NESα3	TubA1a,TubA1b,TubA1c, TubA3a, TubA4a, TubA8	IVSSITASLRF	*GATCCAATTGTTTCTTCTATTACTGCTCTTAGATTTTAGGAGCT*	*CCTAAAATCTAAGAGAAGCAGTAATAGAAGAAACAATTG*	H.s. alpha	*BamHI - SacI*	*pCP60*	N.t. BY-2
NESα3	TubA1a,TubA1b,TubA1c, TubA3a, TubA4a, TubA8	IVSSITASLRF	*GTACCCCGGGATTGTGTCCTCCATCACTGCTTCCCTGAGATTTTAG*	*GATCCTAAAATCTCAGGGAAGCAGTGATGGAGGACACAATCCCGGG*	H.s. alpha	*BamHI - BsrGI*	*pEGFP-C3*	H.s. U-2 OS
NESα3	TUA3-5	IISSLTTSLRF	*GATCCAATCATTTCATCCTTGACAACATCTTTG*	*AGCTTCTAAAACCTCAAAGATGTCAAGGATGAAATGATTG*	A.t. alpha	*BamHI - HindIII*	*0029 pGreen*	N.t. BY-2
NESα3	TUA3-5	IISSLTTSLRF	*GTACCCCGGGATCATTTCATCCTTGACAACATCTTTGAGGTTTTAG*	*GATCTAAAACCTCAAAGATGTTGTCAAGGATGAAATGATCCCGGG*	A.t. alpha	*BamHI - BsrGI*	*pEGFP-C3*	H.s. U-2 OS
NESα4	TUA1, TUA3-5	LRFDGAINV	*ATGAGTAAAGGAGAAGAAAC*	*TTACACATTGATGGCACCGTCAAATCTTAGGTATTTGTATAGTTCATC*	A.t. alpha	*XbaI - BamHI*	*pCP60*	N.t. BY-2
NESα4	TUA1, TUA3-5	LRFDGAINV	*GTACCCCGGGTTGCGGTTCGACGGAGCCATCAACGTGTAG*	*GATCCTACACGTTGATGGCTCCGTCGAACCGCAACCCGGG*	A.t. alpha	*BamHI - BsrGI*	*pEGFP-C3*	H.s. U-2 OS
NESβ1	TUB1, TUB2-3, TUB5, TUB9, TUBB2A, Tub4A, Tubb4B, Tubb6, TUBB8/	LQLERINV	*ATGAGTAAAGGAGAAGAAAC*	*TTAAACATTGATACGTTCGAGCTGAAGGTATTTGTATAGTTCATCCATG*	A.t. beta	*XbaI - BamHI*	*pCP60*	N.t. BY-2
NESβ1	TUB1, TUB2-3, TUB5, TUB9, TUBB2A, Tub4A, Tubb4B, Tubb6, TUBB8/	LQLERINV	*GTACCCCGGGTTGCAGCTGGAGAGAATCAACGTGTAG*	*GATCCTACACGTTGATTCTCTCCAGCTGCAACCCGGG*	H.s. beta	*BamHI - BsrGI*	*pEGFP-C3*	H.s. U-2 OS
NESβ2	TUB1, TUB2-3, TUB4, TUB5, TUB6, TUB7, TUB8, TUB9, TUBB2A, TUBB3,	ICFRTLKL	*ATGAGTAAAGGAGAAGAAAC*	*TTAGAGTTTGAGAGTGCGGAAACAGATGTATTTGTATAGTTCATCCATG*	A.t. beta	*XbaI - BamHI*	*pCP60*	N.t. BY-2
NESβ2	TUB1, TUB2-3, TUB4, TUB5, TUB6, TUB7, TUB8, TUB9, TUBB2A, TUBB3,	ICFRTLKL	*GTACCCCGGGATCTGCTTCCGCACCCTGAAGCTGTAG*	*GATCCTACAGCTTCAGGGTGCGGAAGCAGATCCCGGG*	H.s. beta	*BamHI - BsrGI*	*pEGFP-C3*	H.s. U-2 OS
NESβ3	TUB1, TUB2-3, TUB4, TUB5, TUB6, TUB7, TUB8, TUB9	LNSDLRKLAV	*ATGAGTAAAGGAGAAGAAAC*	*TTACACGGCGAGTTTCCTAAGGTCAGAGTTGAGGTATTTGTATAGTTCATCCAT*	A.t. beta	*XbaI - BamHI*	*pCP60*	N.t. BY-2
NESβ3	TUB1, TUB2-3, TUB4, TUB5, TUB6, TUB7, TUB8, TUB9	LNSDLRKLAV	*GTACCCCGGGCTGAACAGCGACCTGCGCAAGCTGGCGGTGTAG*	*GATCCTACACCGCCAGCTT GCGCAGGTCGCTGTTCAGCCCGGG*	A.t. beta	*BamHI - BsrGI*	*pEGFP-C3*	H.s. U-2 OS

b) Site-directed mutagenesis of NES sequences

Mutated sequences labeled as		AA sequence	Forward oligo	Reverse oligo	Sequence origin	Restriction sites	Expression vectors	For expression in:
mut1NESα2		FGSLLLERLSV	*CTATACAAATACTTCGGTTCTTTACTGTTG*	*CAACAGTAAAGAACCGAAGTATTTGTATAG*	A.t. alpha	*XbaI - BamHI*	*0129 pGreen*	N.t. BY-2
mut2NESα2		LGSLASERLSV	*CTATACAAATACTCAGGTTCTTTAGCTTCTGAGCGCTTGTC*	*GACAAGCGCTCAGAAGCTAAAGAACCTGAGTAT*	A.t. alpha	*XbaI - BamHI*	*0129 pGreen*	N.t. BY-2
mutNESβ2		SCARTLKL	CTATACAAATACAGCTGTGCCCGCACTCTC	*GAGAGTGCGGGCACAGCTGTATTTGTATAG*	A.t. beta	*XbaI - BamHI*	*0029 pGreen*	N.t. BY-2
mutNESβ3		SNSDARKLAV	CTATACAAATACAGCAACTCTGACGCTAGGAAACTC	*GAGTTTCCTAGCGTCAGAGTTGCTGTATTTGTAG*	A.t. beta	*XbaI - BamHI*	*0029 pGreen*	N.t. BY-2

c) Site-directed mutagenesis of tubulins

Mutated sequences labeled as		AA sequence	Forward oligo	Reverse oligo	Sequence origin	Restriction sites	Expression vectors	For expression in:
GFP-AtTUB6-mutNESβ2		SCARTLKL	*CCTTTACGACAGCTGTGCTAGAACACTTAAG*	*CTTAAGTGTTCTAGCACAGCTGTCGTAAAGG*	A.t. beta	*XhoI - SpeI*	*XVE*	N.t. BY-2
GFP-AtTUB6-mutNESβ3		SNSDARKLAV	*CCCGGGTCAGAGCAACTCTGATGCGAGGAAGCTC*	*GAGCTTCCTCGCATCAGAGTTGCTCTGACCCGGG*	A.t. beta	*XhoI - SpeI*	*XVE*	N.t. BY-2

Supplementary information 1
d) Map of pCP60 binary vector

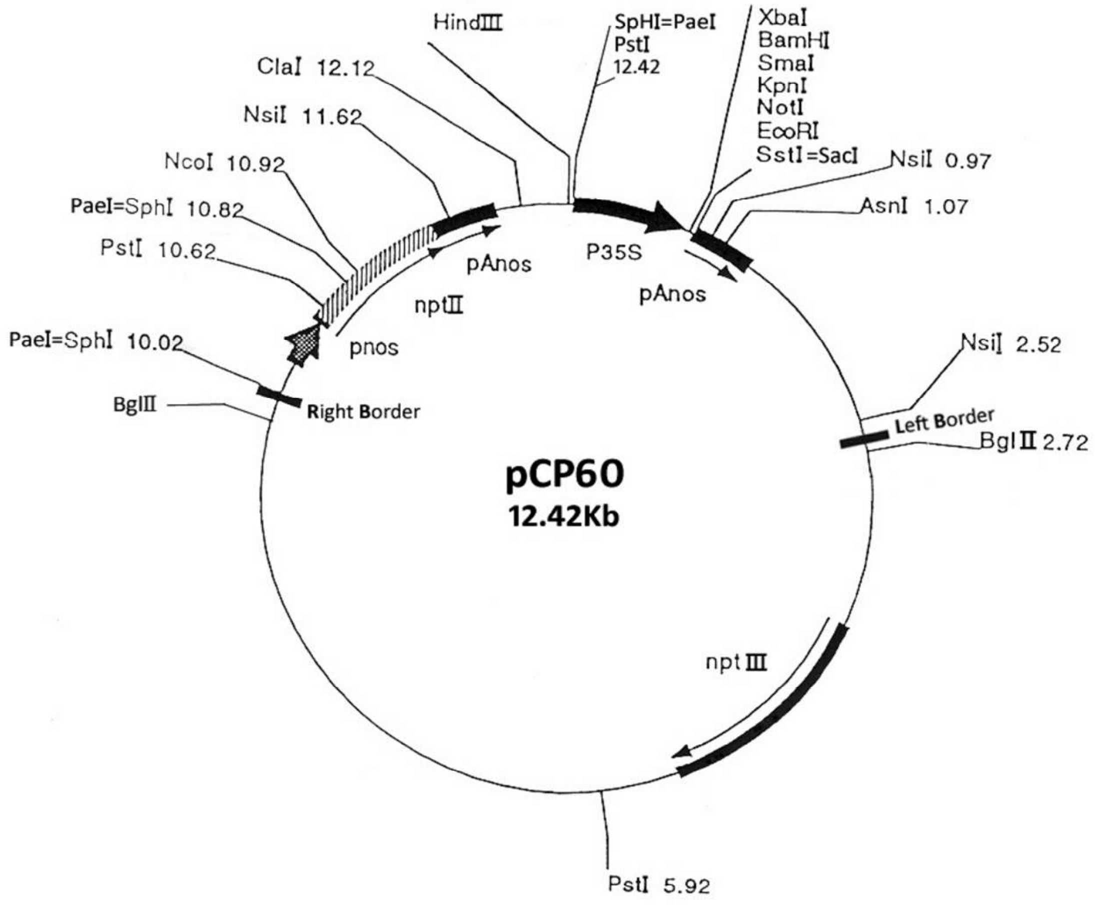

Plasmid name: **pCP60**
Plasmid size: **12.42 Kb**
Constructed by: C. Coronado & P. Ratet
Construction date: 1992
Comments/References: Expressing vector derived from pBin19. The restriction sites between the P35S and the nospA are unique in the vector.

Supplementary information 1
e) details of pCP60 T-DNA

pCP60 T-DNA region

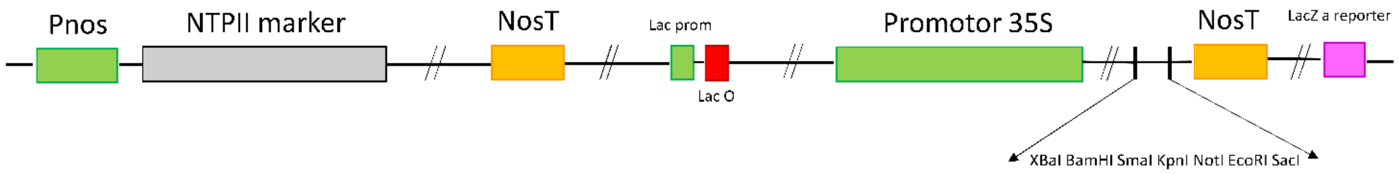

Supplementary information 2:

Expression of mutated tubulins in tobacco BY-2 cells

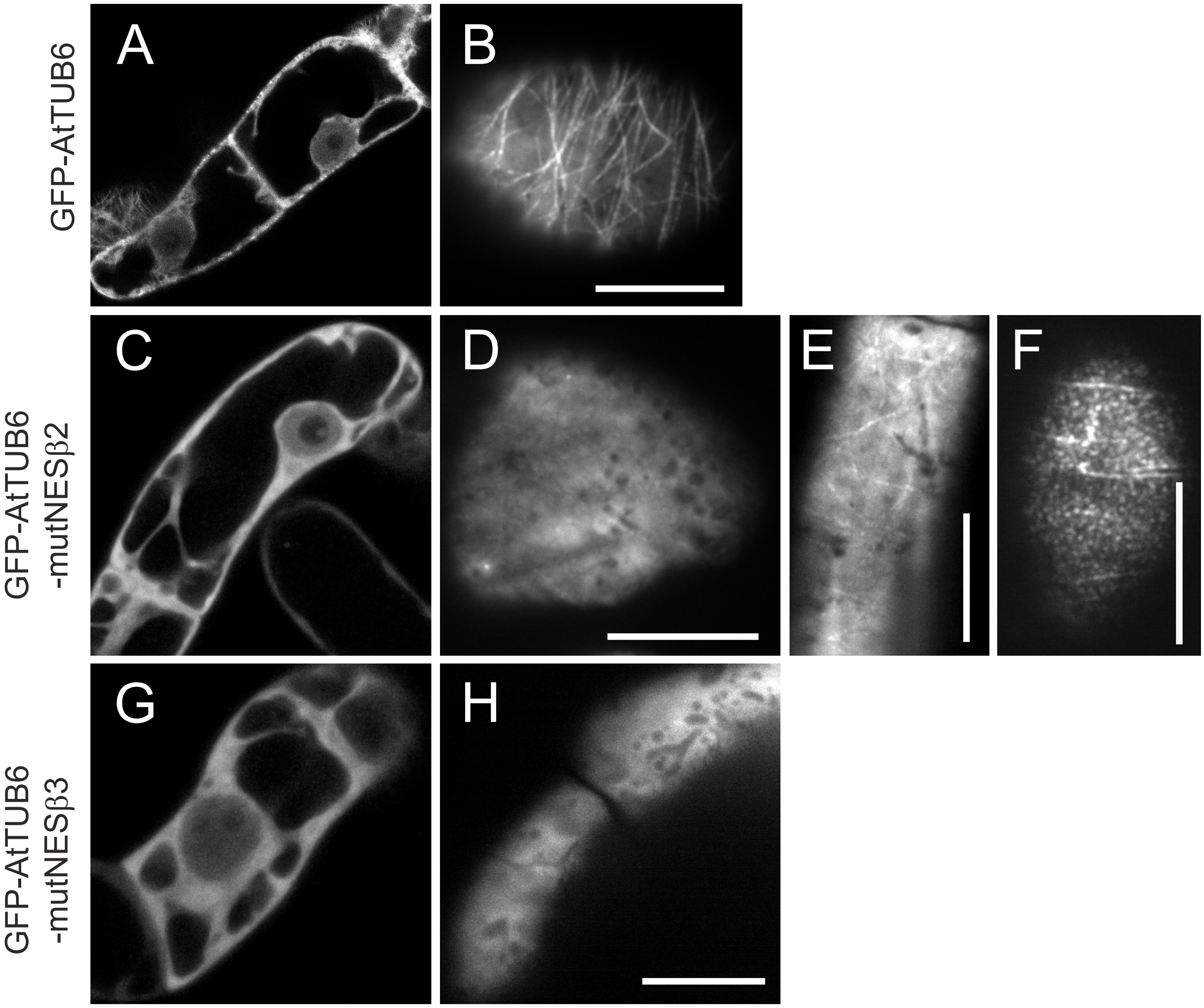

Microtubules localization in human U-2 OS and tobacco BY-2 cells in interphase.

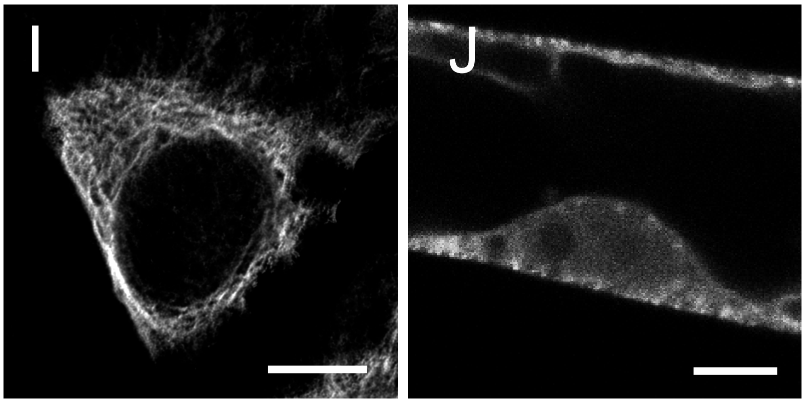

Supplementary information 3

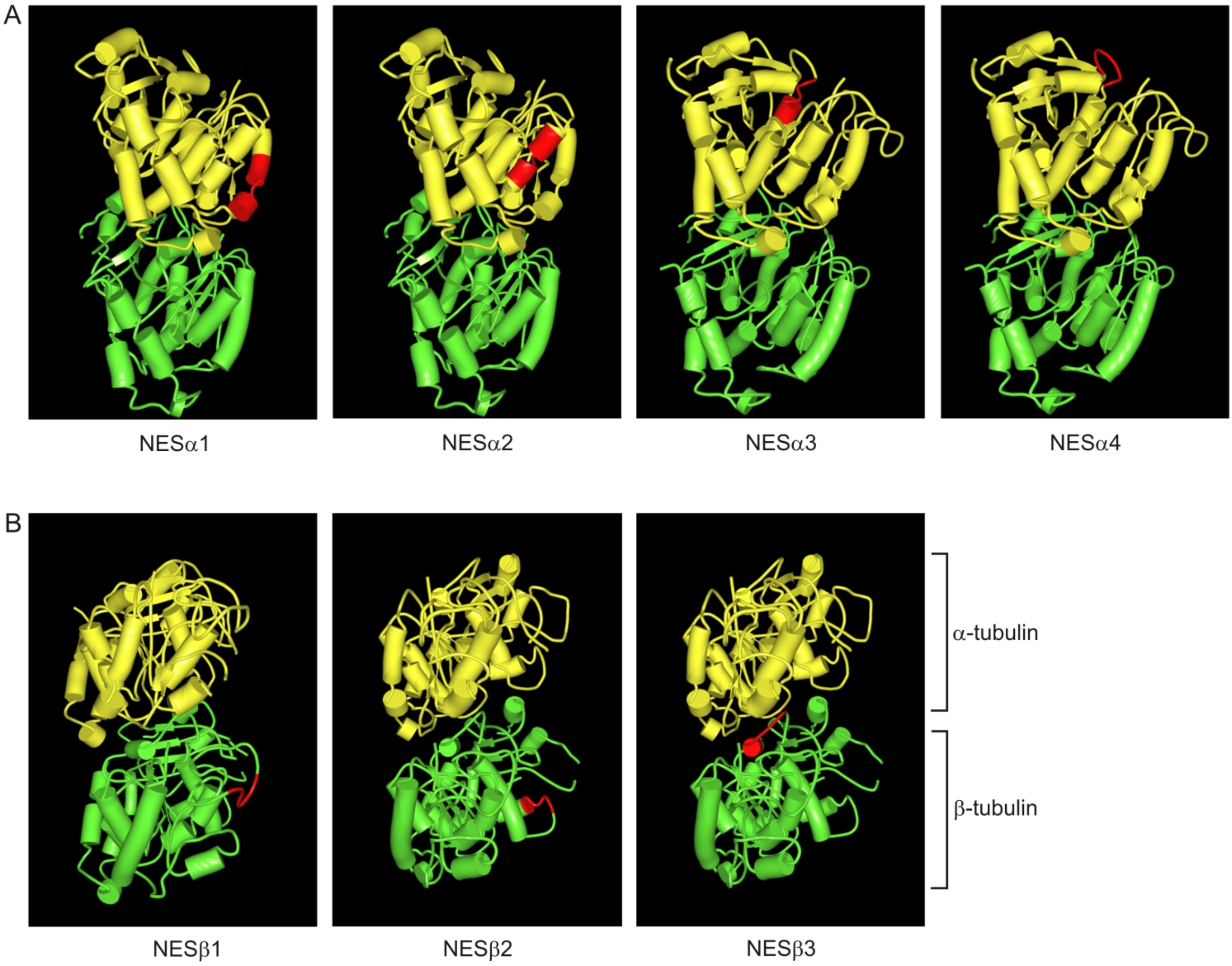

Supplement: Supplementary file 1 — Supplementary information [file 41598_2019_42056_MOESM1_ESM.pdf]
